# Pharmaceutical Applications of Metal Complexes and Derived Materials

**DOI:** 10.3390/pharmaceutics17111373

**Published:** 2025-10-24

**Authors:** Pedro Ivo da Silva Maia

**Affiliations:** Núcleo de Desenvolvimento de Compostos Bioativos (NDCBio), Universidade Federal do Triângulo Mineiro, Av. Dr. Randolfo Borges 1400, Uberaba 38025-440, MG, Brazil; pedro.maia@uftm.edu.br

## 1. Introduction

Medicinal Inorganic Chemistry has demonstrated incomparable potential in the design and development of metal complexes and related materials for a wide spectrum of pharmaceutical applications, including those for therapeutic, diagnostic, and theranostic purposes [1,2,3]. The strategic coordination of bioactive organic molecules with metal centers is key to overcoming the numerous limitations inherent in currently used chemotherapies, such as their high systemic toxicity, reduced efficacy during chronic disease phases, and cellular or microbial resistance [4,5]. Significant efforts have been dedicated to advancing precise treatment and molecular imaging technologies for cancer and parasitic diseases like leishmaniasis based on the outstanding properties of metal complexes, which often show unexpected behaviors in biological environments, where they can exhibit diverse mechanisms of action [1,3,6]. Key properties of these compounds include, but are not limited to, luminescence, magnetic behavior, participation in redox processes, DNA cleavage ability, and the capacity for radiolabeling with metal radioisotopes [1,2,3,6,7,8]. Nonetheless, despite considerable progress over the past two decades, many challenges remain in this field.

In this context, the present Special Issue is dedicated to the development of novel biologically active metal-based compounds and related materials, including nanoclusters, used as pharmacological tools. It includes reports on their synthesis and characterization, radiolabeling of molecules and biomolecules, both in vivo and in vitro pharmacological evaluation, and investigations of their biological targets, providing readers with a comprehensive overview of the applications of metal complexes in the field of Medicinal Chemistry.

## 2. Overview of Published Works

The articles published in this Special Issue span diverse research areas, all unified by a common theme: the medicinal potential of metal-based derivatives. In Contribution (1), a series of gold(I) and silver(I) complexes obtained from hybrid sulfonamide/thiourea ligands were evaluated for their activity against two *Leishmania* strains, as well as on Vero cells. Biological assays revealed that several of these compounds exhibited remarkable leishmanicidal activity, surpassing that of the reference drug Glucantime. Interestingly, the compounds showed differing selectivity between the two *Leishmania* species. Enzymatic studies supported that the compounds have a strong affinity for the Old Yellow Enzyme (OYE) from *Leishmania braziliensis* and can effectively inhibit the enzymatic reaction associated with its activity. Furthermore, a molecular docking analysis revealed that modifications to the peripheral groups of the ligands influence their interactions within the active site of OYE. Taken together, these findings suggest that this class of metal complexes holds promise as a platform for the development of new therapeutic agents for the treatment of leishmaniasis.

In Contribution (2), a gold cluster functionalized with an optimized peptide was developed to target Glioblastoma multiforme (GBM), the most aggressive type of brain tumor. This functionalized cluster efficiently targets GBM cells both in vitro and in vivo. Notably, the uptake of this novel nanomaterial increases the concentration of Fe^2+^ within GBM tumor cells, promoting lipid peroxidation and ferroptosis via a non-canonical mechanism involving the Fenton reaction of Fe^2+^. Additionally, the material demonstrates excellent biosafety and is capable of penetrating the blood–brain barrier.

In Contribution (3), the antiproliferative activity of two tributyltin(IV) derivatives, derived from carboxylate ligands containing an oxoquinoline moiety, was evaluated in vitro against several tumor cell lines, including MCF-7 (human breast adenocarcinoma), HCT116 (human colorectal carcinoma), A375 (human melanoma), 4T1 (mouse breast carcinoma), CT26 (mouse colon carcinoma), and B16 (mouse melanoma). Both compounds exhibited significant cytotoxic activity against A375 cells. Notably, these tributyltin(IV) derivatives induced anchorage-independent apoptosis and reduced adhesion in A375 cells, leading to *anoikis*, a particular form of apoptotic cell death associated with metastasis prevention.

Contributions (4) and (5) employ similar approaches for the development of novel ^99m^Tc-based radiopharmaceuticals. In Contribution (4), a dithiocarbazate molecule was used in radiolabeling studies, whereas Contribution (5) utilized a nonpeptide neurotensin receptor antagonist. Both studies included biodistribution analyses in tumor-bearing mice. Contribution (4) demonstrated that the ^99m^Tc-dithiocarbazate derivative exhibits heterogeneous accumulation in TM1M tumors, but not in the B16-F10 lineage. In Contribution (5), imaging of HT-29 tumor xenografts revealed high NTSR1-specific uptake for one of the radioligands, highlighting its promising potential for SPECT imaging of Neurotensin Receptor-positive tumors. Together, these findings represent significant advances in the field of radiopharmaceutical development.

Finally, in Contribution (6), the cytotoxicity of a series of Pd(II) complexes incorporating triphenylphosphine and thiosemicarbazone ligands was evaluated in both tumor and non-tumor cell lines. Further mechanistic investigations were conducted to assess their effects on tumor cell proliferation, migration, and survival. The results demonstrated that these complexes exhibit both cytotoxic and cytostatic activities, depending on their structural variations, with apoptosis identified as the primary mode of cell death. Remarkably, one of the palladium complexes induced a pronounced increase in late apoptotic cell populations and maintained its cytotoxic efficacy in 3D spheroid models, promoting spheroid disintegration, loss of cell adhesion, and nuclear fragmentation. Overall, the results highlight the potential of the developed Pd(II) complexes as promising anticancer agents capable of overcoming chemoresistance.

## 3. Perspectives

The topics addressed in this Special Issue are of great scientific and medical interest and will undoubtedly continue to be so. However, this Guest Editor believes that three key areas still require significant progress. First, due to the lack of interest from the pharmaceutical industry, there remains an urgent need for effective drugs to treat neglected diseases, particularly parasitic infections. Second, the development and evaluation of compounds incorporating alternative radioisotopes for PET imaging and radiotherapy, such as ^177^Lu (β^−^, t_1_/_2_ = 6.7 days), ^89^Zr (β^+^, t_1_/_2_ = 78.4 h), and ^198^Au (E_β- max_ = 0.96 MeV E_γ_ = 412 keV, t_1/2_ = 2.7 days), is still limited. More research is needed in this area, especially regarding their conjugation to biomolecules with high specificity for disease targets. Finally, despite the significant advances showcased in the contributions to this Special Issue, metal-based compounds still face a degree of prejudice and skepticism that hinders their progression to clinical trials. Nevertheless, given the steady and promising developments in Medicinal Inorganic Chemistry in recent years, broader clinical translation and acceptance of these compounds are expected in the near future.
